# How the Norwegian population was affected by non-pharmaceutical interventions during the first six weeks of the COVID-19 lockdown

**DOI:** 10.1177/14034948211027817

**Published:** 2021-07-12

**Authors:** Silje Mæland, Ragnhild Bjørknes, Stine Lehmann, Gro Mjeldheim Sandal, William Hazell, Åsgeir Kjetland Rabben, Øystein Vedaa, Jens Christoffer Skogen, Lars Thore Fadnes

**Affiliations:** 1 Department of Global Public Health and Primary Care, Faculty of medicine, University of Bergen, Norway; 2 Research Unit for General Practice in Bergen, The Norwegian Research Centre, NORCE, Norway; 3 Department of health promotion and development, Faculty of psychology, University of Bergen, Norway; 4 Department of Psychosocial Science, Faculty of psychology, University of Bergen, Norway; 5 Bergen Municipality, Norway; 6 Department of Health Promotion, Norwegian Institute of Public Health, Norway; 7 Department of Mental Health, Norwegian University of Science and Technology, Norway; 8 Department of Research and Development, St Olav’s University Hospital, Norway; 9 Voss District Psychiatric Hospital, NKS Bjørkeli, Norway; 10 Department of Public Health, Faculty of Health Sciences, University of Stavanger, Norway; 11 Alcohol and Drug Research Western Norway, Stavanger University Hospital, Norway; 12 Centre for Evaluation of Public Health Measures, Norwegian Institute of Public Health, Norway; 13 Bergen Addiction Research, Department of Addiction Medicine, Haukeland University Hospital, Norway

**Keywords:** Non-pharmaceutical interventions, COVID-19, public health, pandemic, mitigation, suppression, social isolation, social distancing, health-care seeking, health-care service utilisation

## Abstract

**Aims:**

The aim of this study was to examine how the Norwegian general adult population was affected by non-pharmaceutical interventions during the first six weeks of the COVID-19 lockdown. We assessed quarantine, symptoms, social distancing, home office/school, work status, social contact and health-care contact through digital access and knowledge.

**Methods:**

A cross-sectional survey was performed of 29,535 adults (aged 18–99) in Norway after six weeks of non-pharmaceutical interventions in March/April 2020.

**Results:**

Most participants found the non-pharmaceutical interventions to be manageable, with 20% of all adults and 30% of those aged <30 regarding them as acceptable only to some or a limited degree. Sixteen per cent had been quarantined, 6% had experienced symptoms that could be linked to COVID-19 and 84% practiced social distancing. Eleven per cent reported changes in the use of health and social services. Three-quarters (75%) of those who had mental health or physiotherapy sessions at least monthly before the pandemic reported a reduction in their use of these services. A substantial reduction was also seen for home nursing, hospital services and dentists compared to usage before the non-pharmaceutical interventions. Immigrants were more likely to experience a reduction in follow-up from psychologists and physiotherapy. With regard to the use of general practitioners, the proportions reporting an increase and a reduction were relatively equal.

**Conclusions:**

**The non-pharmaceutical interventions were perceived as manageable by the majority of the adult general population in Norway at the beginning of the COVID-19 pandemic. A substantial proportion of adults <30 years old experienced difficulties with social distancing, and those >70 years old lacked the digital tools and knowledge. Further, immigrant access to health services needs monitoring and future attention.**

## Introduction

The World Health Organization (WHO) declared COVID-19 a pandemic on 11 March 2020. In the absence of a vaccine for COVID-19, many countries have used non-pharmaceutical interventions (NPIs) to control the spread of the infection [1]. NPIs aim to reduce contact frequency among individuals, thus limiting transmission of the virus [1]. Mitigation aims to slow down the speed of human-to-human transmission of the virus, with the protection of vulnerable groups. Modelling shows that the mitigation strategy can reduce the burden on the health-care system by two thirds and halve the number of deaths [1].

In the absence of vaccines and drugs, the NPIs in Norway comprised a mix between public orders and recommendations. Schools, universities, all public and the majority of private businesses were ordered to close physically, and working from home was mandatory when possible. Services such as hairdressers, shops and activity arenas for sport and culture were ordered to close. Health and social services were given specific instructions on when and where services could be provided to the population. General practitioners (GPs), physiotherapists, psychologists and dentists underwent statutory shutdowns or redirection of their services to digital platforms [2], thus increasing the demand for digital access and knowledge about use in the general adult population. Many Norwegian GPs reported a quick and smooth shift to digital consultations [3], and separate community airway clinics were set up. The following NPIs were recommended: quarantine after travelling abroad, travel restrictions, restricted use of public areas and public transport and general social distancing, limiting social contact to those who lived in the same household. Research on the psychological consequences of similar quarantine measures to prevent infection during prior epidemics has shown an increased incidence of symptoms, including insomnia, concentration problems, worry, anxiety and depression [4]. Subsequently, maintaining a well-functioning health and welfare system is important during a pandemic such as COVID-19.

During the COVID-19 pandemic, there has been uncertainty related to the number of infected individuals. For several of the initial months of the pandemic, access to testing equipment and lab capacity to provide accurate numbers were sparse [5], and only people with severe symptoms and those in high-risk groups with milder symptoms were tested. As a result, strict NPIs were imposed on individuals with an uncertain infection status, and this may have influenced their health-care-seeking behaviour. Additionally, rearranging resources in the health-care services may have altered the population’s health-care-seeking behaviour for non-COVID-19-related health problems. Thus, there is great uncertainty related to the consequences to public health due to changes in access to/use of health-care services in the first period of COVID-19 NPIs. Therefore, this study investigated the general population’s experiences of NPIs in Norway and possible changes in health-care service utilisation during the first six weeks of COVID-19.

### Aims

We aimed to examine how the general adult population (aged 18–99 years) were affected by the NPIs in their everyday life during the first six weeks of the COVID-19 lockdown. We assessed quarantine, COVID-19-related symptoms, social distancing, working from home, home schooling, work status, how easy it was to maintain social contact with others through digital access and how this knowledge was perceived, and how this affected health-care contact and follow-up. We examined socio-demographic factors and the risk of the reduction in use of health services.

## Method

### Participants and data collection

A random sample of 81,170 individuals from a total of 224,000 adult inhabitants (aged 18–99 years) in the city of Bergen, Western Norway, were invited to participate in a study investigating the consequences of COVID-19 and NPIs. The sample was representative of the general population with regard to age and sex. Individuals invited to participate were drawn from the Norwegian contact register through the Norwegian Digitalisation Agency. An electronic web-based survey was distributed by email on 15 and 16 April 2020. Two reminders were sent – one by text message and one by email – and the survey was closed on 30 April 2020. In all, 29,535 (36%) individuals consented to participate. It took participants about 15–20 minutes to complete the survey.

### Measurements included in the survey

Demographic information included age, sex, educational level, total household income, household size, employment status before the COVID-19 outbreak, change in employment status after the COVID-19 outbreak and if self or parents had immigrated to Norway.

The outcome variables used in this study were self-reported responses on: been ill with suspected, possible or confirmed COVID-19 during the past four weeks; living in the same household as someone with suspected, possible or confirmed COVID-19 during the past four weeks; and access to digital tools and use of different health and social services (e.g. GP, homecare, psychologist, physiotherapist). Questions specifically related to the COVID-19 NPIs during the past four weeks included: quarantine, social distancing, working from home and/or home schooling; to what degree access to and knowledge of digital equipment influenced contact with others; and whether the NPIs were difficult to handle. The feasibility of the NPIs were assessed using a three-point scale (feasible to a large, some or a limited degree).

### Statistical analyses

The analyses were performed with Stata SE v16 (StataCorp, College Station, TX), and graphical presentation of the figure was done with SankeyMATIC [6]. There was a lower response rate among younger participants and men compared to women and older age groups. For many of the analyses, we have stratified by age groups (⩾18–<30, ⩾30–<40, ⩾40–<50, ⩾50–<60 and ⩾70). Thus, we used inverse probability weights based on age and sex to balance the sample according to the background population distribution. The weights were calculated using binomial regression models, with mean weights of 1.0 with a standard deviation of 0.25. Weighted estimates for the total sample are presented with 95% confidence intervals (CI). Descriptive analyses are presented in cross-tables as medians with corresponding 25th and 75th percentiles. Chi-square tests were used to test statistical significance of change. Change in health services used is presented in Sankey diagrams, showing change from before to during the COVID-19 outbreak and related interventions. To assess the odds of reduction in frequency of follow-up from various health-care providers based on various socio-demographic indicators, logistic regression was conducted. Odds ratios (OR) are presented with 95% CI.

The study was approved by the Norwegian Regional Committee for Ethics in Medical Research (REK 2020/131560). All participants provided written informed consent before responding to the emailed survey, and confidentiality and the right to withdraw from the study were assured. The study conforms with the ethical principles outlined in the Declaration of Helsinki.

## Results

Among the 29,535 participants, 56% were women with a median age of 50 years (range 36–63 years). Sixty-four per cent had more than three years of university or senior high school education, 93% were born in Norway, 87% had a medium to high household income (>NOK250k/person), 7% were students and 59% were employed. A total of 54% lived together with one or two other people (Table I).

**Table I.** Demographic characteristics for the participants in each age group.

Category/age (years)	18–29	30–39	40–49	50–59	60–69	70+	Total
*n* (response rate)	4448 (22%)	5068 (31%)	5461 (39%)	5843 (46%)	4798 (52%)	3917 (42%)	29,535 (36%)
Females	2758 (62%)	2976 (59%)	3097 (57%)	3268 (56%)	2447 (51%)	1851 (47%)	16,397 (56%)
Primary/secondary school	1622 (49%)	944 (23%)	1141 (24%)	1930 (36%)	1866 (42%)	1681 (46%)	9184 (36%)
University/high school	1715 (51%)	3200 (77%)	3579 (76%)	2556 (63%)	2632 (58%)	1949 (54%)	16,418 (64%)
Born in Norway	4104 (92%)	4383 (86%)	5002 (92%)	5581 (96%)	4650 (97%)	3840 (98%)	27,560 (93%)
Household income in NOK (adjusted per person^a^)							
0–250,000	1054 (36%)	537 (13%)	479 (11%)	377 (8%)	251 (7%)	383 (13%)	3081 (13%)
250,000–500,000	1116 (38%)	1977 (50%)	2301 (51%)	1842 (38%)	1380 (36%)	1441 (50%)	10,057 (44%)
>500,000	740 (25%)	1475 (37%)	1700 (38%)	2596 (54%)	2230 (58%)	1051 (37%)	9792 (43%)
Number of people in household							
1	477 (14%)	617 (15%)	514 (11%)	880 (17%)	1236 (29%)	1463 (43%)	5187 (21%)
2	1176 (36%)	886 (22%)	615 (13%)	1620 (31%)	2210 (51%)	1561 (46%)	8068 (32%)
3–4	1225 (37%)	1965 (48%)	2316 (50%)	2146 (42%)	800 (18%)	331 (10%)	8783 (35%)
⩾5	433 (13%)	650 (16%)	1211 (26%)	510 (10%)	84 (2%)	51 (1%)	2939 (12%)
Employed/in work	2206 (50%)	3598 (96%)	4243 (78%)	4654 (80%)	2524 (53%)	230 (6%)	17,455 (59%)
Student	1607 (36%)	257 (5%)	105 (2%)	32 (1%)	7 (0%)	3 (0%)	2011 (7%)
Lacking access to							
Internet	154 (3%)	71 (1%)	80 (1%)	60 (1%)	32 (1%)	18 (0%)	415 (1%)
Web camera	87 (2%)	117 (2%)	116 (2%)	152 (3%)	181 (4%)	232 (6%)	885 (3%)
Laptop/computer/tablet	39 (1%)	65 (1%)	51 (1%)	57 (1%)	25 (1%)	23 (1%)	260 (1%)
Smart phone	11 (0%)	10 (0%)	10 (0%)	26 (0%)	19 (0%)	66 (2%)	142 (0%)
Necessary software	87 (2%)	96 (2%)	122 (2%)	127 (2%)	121 (3%)	134 (3%)	687 (2%)
One or more of the above	258 (6%)	245 (5%)	259 (5%)	279 (5%)	240 (5%)	274 (7%)	1555 (5%)

a Household income is divided with a person index calculated as 1 for first adult, 0.7 for other adults and 0.5 for a child.

In the sample, 6% (95% CI 5–8%) reported suspected COVID-19 symptoms, 4% (95% CI 3–5%) lived with people with suspected COVID-19 symptoms, 16% (95% CI 14–19%) had been quarantined and 84% (95% CI 83–85%) reported that they practiced social distancing (Table II). In total, 19% of people in quarantine had symptoms that could be linked to COVID-19, while 11% had close contact with others with symptoms. A total of 7% (95% CI 5–9%) of participants had lost their job either temporarily or permanently. The NPIs were experienced to be acceptable among 76%, but 11% perceived them as difficult. People <30 years of age experienced adhering to the NPIs as difficult more often than people aged >70 years (OR=6.4; 95% CI 5.2–7.8). Most (79%) respondents reported having received sufficient information. Social distancing was considered as the most challenging of the NPIs (50% of respondents), followed by closed services related to culture, sports and schools/kindergartens (18%, 17% and 17% of respondents, respectively). Among people aged >70 years, 27% lacked knowledge in the use of digital tools, while 10% lacked access to tools such as a web camera or the necessary software. Difficulties keeping in touch with family, friends and services due to lack of digital equipment or skills was reported by 31% of the respondents (35% aged >70 years and 22% aged <30 years).

**Table II.** Number and percentage in each age group experiencing COVID-19-related consequences (total estimates are weighted).

	18–29	30–39	40–49	50–59	60–69	⩾70	All
Quarantined	1251 (21%)	887 (16%)	715 (15%)	705 (14%)	587 (15%)	736 (17%)	16% (14–19)
COVID-19 symptoms	448 (8%)	448 (8%)	361 (8%)	304 (6%)	140 (4%)	92 (2%)	6% (5–8)
Lived with people with COVID-19 symptoms	374 (6%)	274 (5%)	232 (5%)	215 (4%)	86 (2%)	52 (1%)	4% (3–5)
Distancing from others	4984 (85%)	4655 (85%)	3995 (83%)	4214 (83%)	3299 (84%)	3614 (81%)	84% (83–85)
Working from home/home schooling	3654 (63%)	3326 (61%)	3040 (63%)	2626 (52%)	1221 (31%)	185 (4%)	51% (39–62)
Made redundant (temporarily/permanent)	513 (12%)	401 (8%)	362 (7%)	420 (7%)	225 (5%)	20 (1%)	7% (5–9)
Reduced contact due to							
Lack of digital equipment access	339 (9%)	326 (7%)	383 (7%)	424 (8%)	359 (8%)	349 (10%)	8% (7–8)
Lack of knowledge in use of tools	135 (4%)	257 (6%)	476 (9%)	821 (15%)	882 (19%)	984 (27%)	11% (6–16)
NPIs perceived acceptable							
To large degree	2843 (70%)	3681 (76%)	4356 (82%)	4870 (85%)	4068 (86%)	3127 (82%)	76% (71–82)
To some degree	1146 (28%)	1076 (22%)	895 (17%)	766 (13%)	530 (11%)	454 (12%)	18% (14–23)
To limited degree	77 (2%)	76 (2%)	62 (1%)	103 (2%)	134 (3%)	241 (6%)	2% (1–3)
NPIs perceived difficult	750 (23%)	698 (18%)	456 (10%)	361 (7%)	209 (5%)	116 (4%)	11% (7–15)

NPI: non-pharmaceutical interventions.

**Figure 1.**
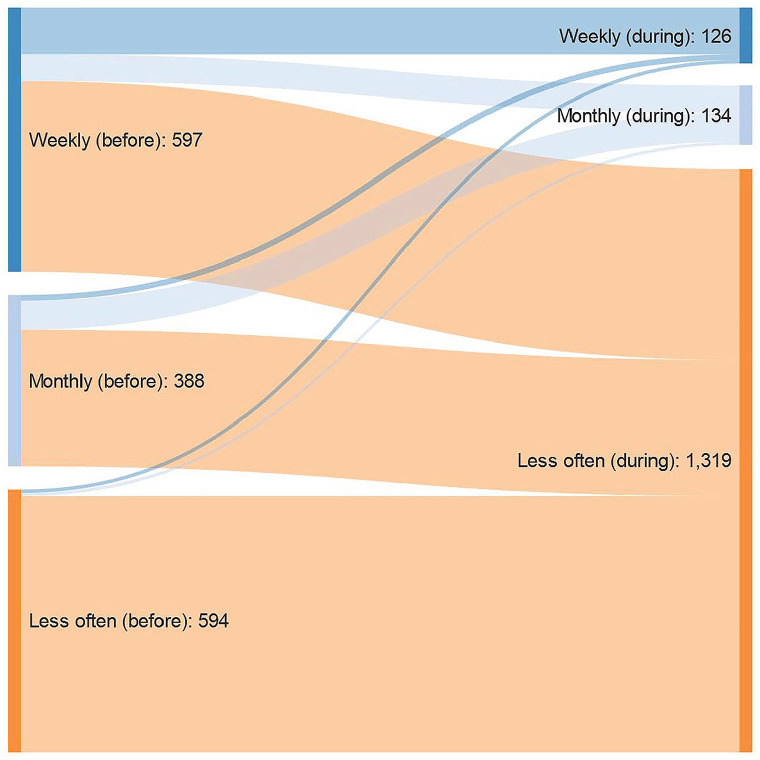
Change in use of health services from home psychologists or physiotherapy (left side) to during period of the COVID-19 pandemic (right side) among those reporting change in health or social services and using the respective health services before the pandemic. Weekly follow-up during the pandemic is labelled with deep blue, monthly follow-up during the pandemic is labelled with light blue, and less frequent follow-up during the pandemic is labelled with orange. *p<0.001 for all changes.

There was a substantial reduction in the use of psychologist and physiotherapist services (51% of those who used services during last month and reported a change in any service reported a reduction, and 2% reported an increase in frequency; *p*<0.001). Among those who had received weekly or monthly treatment sessions prior to the COVID-19 pandemic and reported a change in any services, 75% reported less than monthly follow-up from these services during the outbreak and the NPIs (Figure 1). Regarding health-care utilisation, 11% reported changes in use of health and social services in the period with NPIs compared to before. For GPs, the proportion reporting an increase and a reduction in use were more or less equal (Figure 1, Figures S1–S7 and Supplemental File). For use of home nursing, a larger proportion reported a reduction rather than an increase (16% vs. 4%; *p*<0.01). There was also a reduction in the use of health services from hospital, antenatal and paediatric health clinics and other health services, including dentist services (*p*<0.001). Also, for social services, there was a substantial reduction in use (*p*<0.05).

For use of services from psychologists and physiotherapists and nursing care, immigrants were more likely to report reduction in use (Table III). People living as couples/with two people in the household also experienced a reduction in follow-up during the NPIs (Table III). For use of a GP, no groups were particularly prone to report a reduction in service use.

**Table III.** Logistic regression of odds for reduction in frequency of follow-up based on various socio-demographic indicators.

	General practitioner	Psychologist/physiotherapist	Hospital follow-up	Nursing care	Other health care	Any
Age						
18–30	1	1	1	1	1	1
30–39	0.8 (0.5–1.4)	1.2 (0.7–2.1)	0.9 (0.3–2.6)	0.7 (0.2–3.0)	1.1 (0.5–2.3)	1.0 (0.6–1.5)
40–49	0.7 (0.4–1.2)	1.5 (0.9–2.7)	2.3 (0.7–7.6)	0.4 (0.1–1.9)	1.5 (0.6–3.4)	1.1 (0.7–1.8)
50–59	0.9 (0.5–1.6)	1.3 (0.7–2.3)	0.6 (0.2–1.9)	0.5 (0.1–2.0)	1.7 (0.7–4.5)	1.2 (0.7–1.9)
60–69	0.9 (0.4–1.8)	1.5 (0.7–3.2)	1.0 (0.3–3.5)	0.2 (0.1–1.0)	2.1 (0.6–7.3)	1.1 (0.6–1.9)
⩾70	0.6 (0.3–1.2)	2.1 (0.9–5.3)	1.1 (0.3–4.5)	0.3 (0.1–1.2)	0.8 (0.2–2.7)	0.9 (0.5–1.5)
Sex (female)	1	1	1	1	1	1
Male	1.1 (0.7–1.6)	0.9 (0.6–1.4)	0.5 (0.2–0.9)	1.5 (0.6–3.4)	1.3 (0.6–2.5)	0.8 (0.6–1.0)
Access to digital tools	1	1	1	1	1	1
Lack of access to digital tools	1.0 (0.5–1.8)	2.0 (0.9–4.5)	1.0 (0.3–3.3)	0.4 (0.1–1.6)	0.6 (0.2–1.8)	1.2 (0.7–2.0)
Norwegian	1	1	–	1	1	1
Migrant	0.8 (0.4–1.6)	4.3 (1.3–14)		7.8 (0.9–69)	1.1 (0.3–4.3)	1.9 (1.0–3.7)
Household income						
<250,000	1	1	1	1	1	1
250,000–500,000	0.7 (0.5–1.1)	1.2 (0.7–1.8)	0.8 (0.3–1.8)	1.3 (0.5–3.1)	0.9 (0.5–1.9)	0.9 (0.7–1.3)
>500,000	0.8 (0.5–1.4)	1.5 (0.9–2.6)	0.6 (0.3–1.6)	1.9 (0.7–5.7)	1.2 (0.5–2.5)	1.2 (0.8–1.8)
Number of people in household						
1	1	1	1	1	1	1
2	0.9 (0.5–1.4)	2.0 (1.2–3.5)	0.8 (0.3–2.0)	1.8 (0.7–4.6)	1.6 (0.7–3.8)	1.4 (0.9–2.1)
3–4	1.0 (0.6–1.6)	1.5 (0.9–2.3)	0.6 (0.2–1.4)	2.0 (0.7–5.3)	1.0 (0.5–2.1)	1.1 (0.8–1.6)
⩾5	0.6 (0.3–1.1)	0.8 (0.4–1.5)	0.6 (0.2–1.8)	1.2 (0.3–4.2)	2.1 (0.8–5.5)	0.8 (0.5–1.2)

Data are presented for health care from general practitioner, psychologist and physiotherapist, hospital follow-up, nursing care, other health care and any type of health care as odds ratios with 95% confidence intervals.

## Discussion

This study is based on data from the COVID-19 pandemic in Norway in March/April 2020. The NPIs were largely perceived as manageable. However, for those <30 years of age, one third of the respondents reported social distancing as only manageable to some extent. Many elderly people also experienced challenges, and one third in this group of respondents experienced difficulties keeping in touch with family, friends and health services due to a lack of digital equipment or skills, either themselves or close contacts. This may have caused feelings of loneliness and social isolation within these groups. However, another Norwegian survey with 10,440 participants aged 19–92 years collected in June 2020 and with baseline data on loneliness indicates that overall loneliness was stable or falling. Even so, single individuals and older women reported slightly increased loneliness [7]. In a COVID-19 study from Israel, also with participants aged 18–100 years, loneliness due to the COVID-19 social-distancing policy was the main risk factor for depression and anxiety, and especially their co-morbidity [8]. Previous research on people’s mental health during and following a pandemic suggests that we can expect a considerable increase in anxiety and depression in the general population [4,9]. However, during the first six months of the COVID-19 pandemic, stable levels of mental disorders, suicidal ideation and suicide deaths compared to pre-pandemic levels were found in another Norwegian study [10]. Therefore, continuous investigations and monitoring of adverse effects of NPIs are important. Based on previous studies, there are reasons to expect that the consequences of the NPIs to stop the virus will be magnified among socially vulnerable groups [11,12]. Accordingly, policymakers and mental health practitioners need to stress the importance of safe social interactions [13].

Evidence from the SARS pandemic in Canada demonstrates the added risk to people’s health when the population stops attending health-care services due to concerns about the risk of infection [14]. The largest decrease in availability of services was with psychologists and physiotherapists and with other health services such as dentists. We argue that the closure of health-care services poses a threat to future public health and should thus be avoided as an NPI. An important finding is that immigrants were more prone to have a reduction in follow-up, particularly from psychology and physiotherapy services. Another Norwegian study identified immigrants as more vulnerable to psychological distress during the COVID-19 pandemic [15]. Thus, a reduction in the availability of mental health services may have particularly negative consequences for this group.

There was no clear decrease in the use of GP services, but rather a shift in service use. A Norwegian study found a 47–69% reduction in reported cases for all other infectious diseases in weeks 12–14 of 2020 [16]. This may indicate a decrease in the risk of other infectious diseases due to the NPIs or decreased detection sensitivity, as most resources were redirected to COVID-19 cases [16]. Overall, our findings indicate that GP services were successful shifting quickly to digital tools and platforms to accommodate the changing needs of their patients and society. This is in line with successful pandemic management in Australia where retainment of the functional capability of general practices and the wider primary health-care system ensured the continued provision of regular primary care services to the whole community [17].

Our findings indicate that the Norwegian population experienced little shortage of digital equipment during the pandemic opening for digital consultations, guidance and treatment from health professionals. Figures from the Directorate for e-Health show that the health service had around 20,000 video consultations daily by the end of March 2020 [18]. GPs reported a dramatic increase (more than a tripling) in the use of digital consultations in March and April 2020 [19]. However, this form of contact is not feasible for all conditions or patients [20]. A substantial proportion of those >70 years of age reported a lack of competence in the use of digital tools and platforms, and there may be unintended negative consequences of suboptimal digital solutions. It is therefore important to be aware of which groups in society miss out on the full range of services during a pandemic. One remedy implemented by the Australian government during the COVID-19 pandemic was publicly funded telehealth services to ensure continued access to general practice and other health services [17].

During the summer months of 2020, the NPIs in Norway were eased, but at the beginning of August 2020, the number of COVID-19 infections increased. Thus, the Norwegian government was dependent on trust from the citizens to obey and accept the tightening of the NPIs again. Our results indicate that the majority of citizens perceived the NPIs implemented in March 2020 as manageable, and that enough information was provided by the health authorities. The importance of information and communication has been acknowledged by the Norwegian government as key to future NPIs compliance [21]. In relation to this, our findings that social distancing was perceived as most challenging for those <30 years and that this group was particularly vulnerable to psychological distress [15] are important for monitoring this group closely as the pandemic continues and in the wake of the pandemic. A study conducted in Belgium found that young adults tended to report less living space, occupational activity and social contact and more anxiety, depression and uncertainty than older participants during COVID-19 [22]. Likewise, a study among young Americans aged 18–24 years documented unprecedented and elevated numbers of younger individuals experiencing depression, anxiety and, for some, suicidal ideation [23]. If this added psychological distress can be explained by lockdown conditions and by intolerance to increased uncertainty, this age group might need special attention with regard to mental health services in times when strict NPIs are implemented. Social distancing might be particularly difficult for younger adults, as many live alone. Knowledge about the long-term impact of social isolation for different groups of the population is still scarce.

One strength of this study is its large sample size, allowing analyses with high precision and statistical power. Another strength is that the evolution of the pandemic, policy enactment and measures introduced to counteract this were the same for all respondents in this study. Moreover, the study period coincided with the most invasive implementation of NPIs in Norway until now. One inherent limitation of this study is its cross-sectional design, limiting the possibility for causal inferences. Another limitation of this study is that it relies on self-report and therefore is dependent on the participants’ insights, willingness to report and other response biases. The survey provides a representative sample with regard to age and sex of adults in Bergen municipality, including the second biggest city in Norway with a similar age distribution as the whole country. However, given our finding that 10% of those aged >70 years reported to lack of access to digital equipment and 27% lacked knowledge to keep in touch with others, we can assume that many have not answered our survey in this age group and that this is a much bigger problem. Although web-based questionnaires are a feasible tool for data collection in large population-based epidemiological studies in countries such as Norway [24], the response rate of 36% could be considered relatively low. Only using digital distribution (email and mobile phone) may partly explain this, and for some marginalised groups (e.g. people with limited access and knowledge in use of digital tools, those living in nursing homes, homeless people and people with drug addictions) paper-based response may have been better. Consequently, our findings may not be generalisable to these populations. Due to time constraints, the survey was only available in Norwegian. Thus, the survey is not able to answer how migrants who are not fluent in Norwegian experienced the NPIs. The geographical distribution of the responses shows that we have not succeeded in obtaining responses from inhabitants in rural areas. To compensate for the difference in response rate between groups, we applied inverse probability weights to balance the sample according to the background population distribution. We have no information about the non-responders in relation to NPIs behaviour and adherence to these measures.

## Conclusions

Overall, this study suggests that the initial COVID-19 NPIs were perceived as manageable in everyday life by the majority of the general adult population in Norway. Even so, our results show that a high proportion of the youngest adults (aged <30 years) reported problems with symptoms, quarantine, working from home, home schooling and social distancing. Further, a large proportion of the oldest adults (age >70 years) reported lacking the digital tools and knowledge to keep in touch with others, and the numbers are presumably much bigger, as our data collection was based only on digital responses. It is noticeable that immigrants were less likely to receive regular follow-up by a psychologist/physiotherapist during the first period of the COVID-19 outbreak in Norway. These results indicate the need for close monitoring of these groups during and after the COVID-19 pandemic.

## Supplemental Material

sj-docx-1-sjp-10.1177_14034948211027817 – Supplemental material for How the Norwegian population was affected by non-pharmaceutical interventions during the first six weeks of the COVID-19 lockdown Click here for additional data file. Supplemental material, sj-docx-1-sjp-10.1177_14034948211027817 for How the Norwegian population was affected by non-pharmaceutical interventions during the first six weeks of the COVID-19 lockdown by Silje Mæland, Ragnhild Bjørknes, Stine Lehmann, Gro Mjeldheim Sandal, William Hazell, Åsgeir Kjetland Rabben, Øystein Vedaa, Jens Christoffer Skogen and Lars Thore Fadnes in Scandinavian Journal of Public Health
