# Supplementary material for: How the Norwegian population was affected by non-pharmaceutical interventions during the first six weeks of the COVID-19 lockdown
Source: Scand J Public Health. 2021 Jul 12;50(1):94–101. doi: 10.1177/14034948211027817 (PMC8808225; doi:10.1177/14034948211027817)
Supplement: sj-docx-1-sjp-10.1177_14034948211027817 – Supplemental material for How the Norwegian population was affected by non-pharmaceutical interventions during the first six weeks of the COVID-19 lockdown [file sj-docx-1-sjp-10.1177_14034948211027817.docx]

**Figure S1:** Change in use of health services from general practitioners before (left side) to during period of the COVID-19 pandemic (right side) among those reporting change in health or social services and using the respective health services before the pandemic. Weekly follow-up during the pandemic is labelled with deep blue, monthly follow-up during the pandemic is labelled with light blue, and less frequent follow-up during the pandemic is labelled with orange.


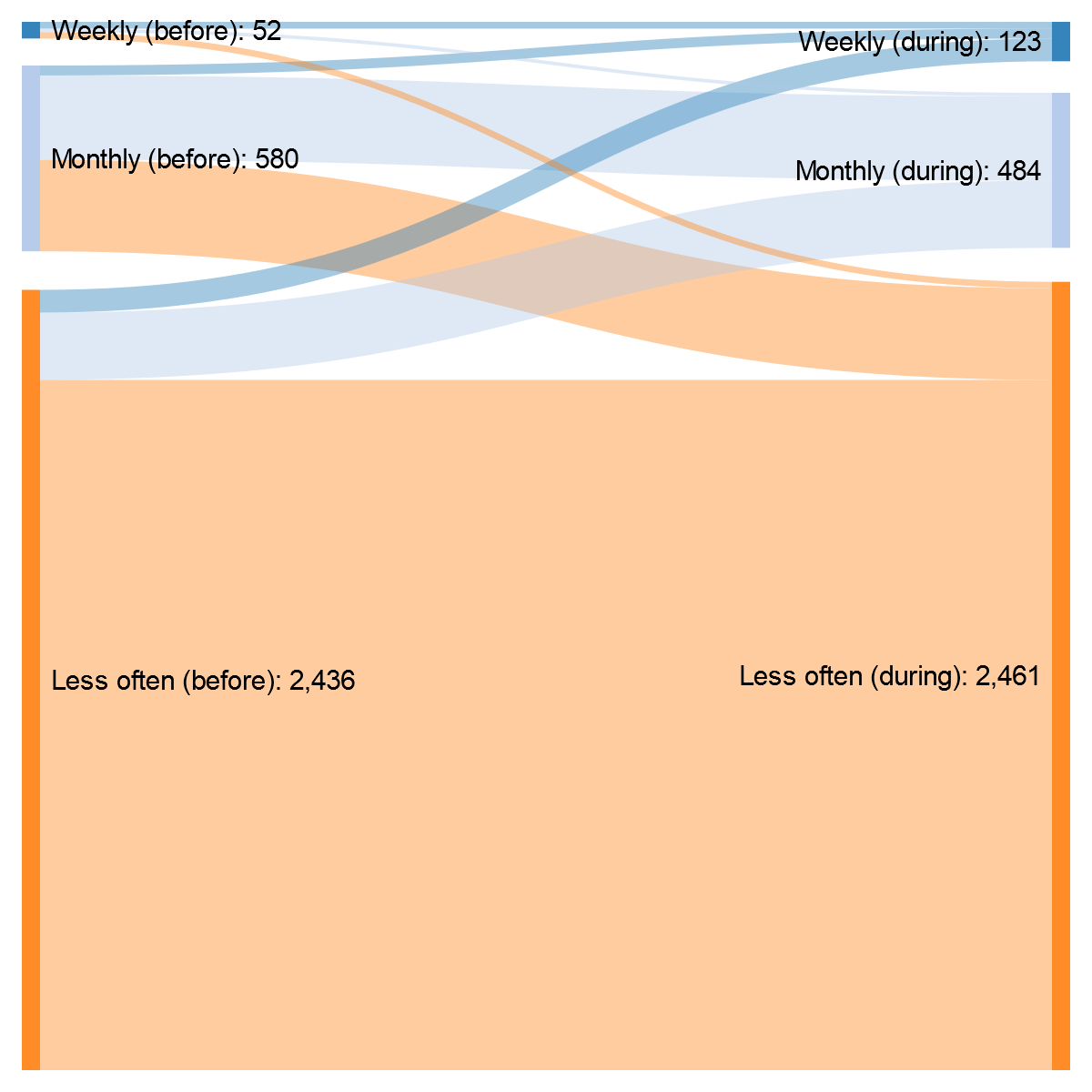


* p < 0.001 for all changes

**Figure S2:** Change in use of health services from home nursing before (left side) to during period of the COVID-19 pandemic (right side) among those reporting change in health or social services and using the respective health services before the pandemic. Weekly follow-up during the pandemic is labelled with deep blue, monthly follow-up during the pandemic is labelled with light blue, and less frequent follow-up during the pandemic is labelled with orange.


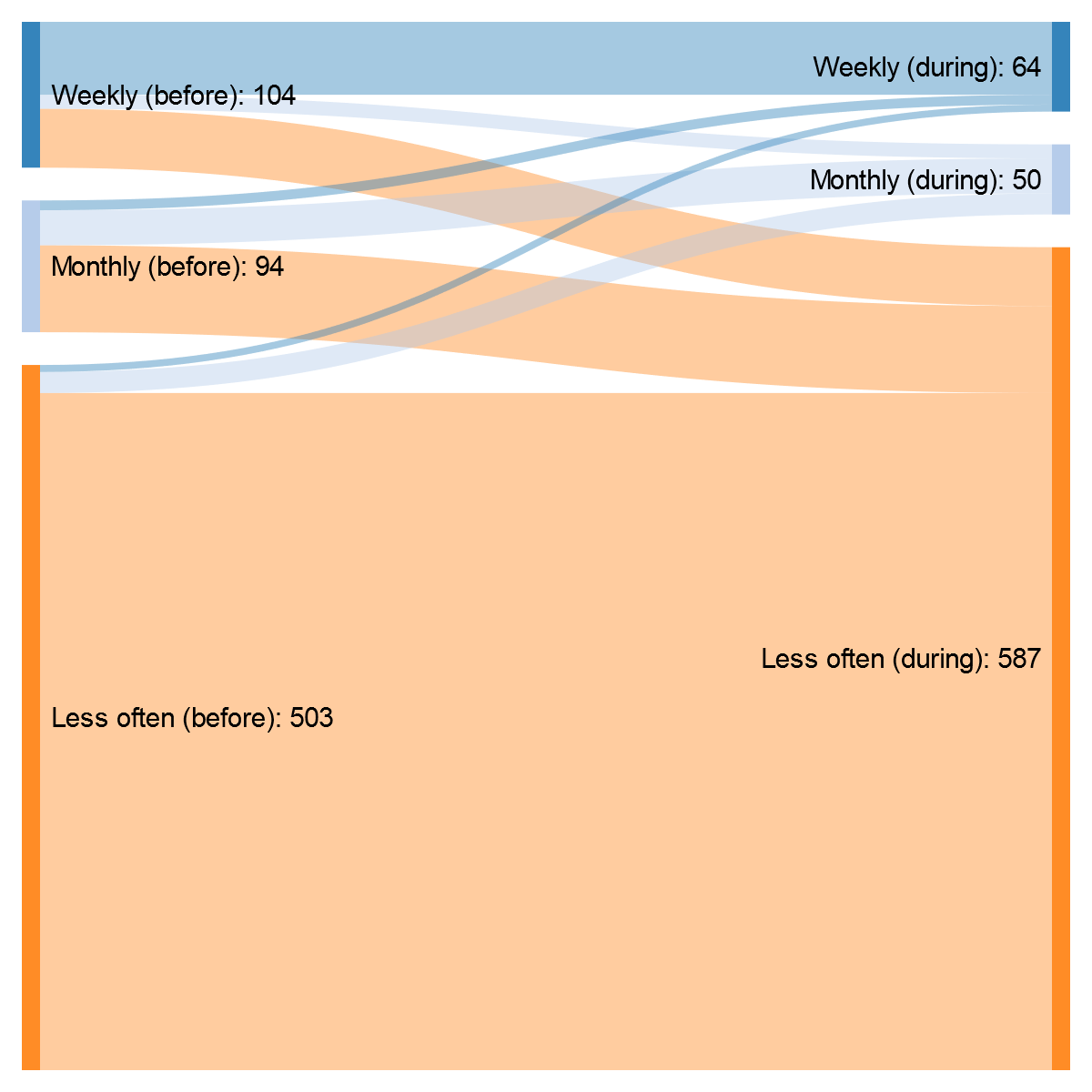


* p < 0.001 for all changes

**Figure S3:** Change in use of hospital-based health services before (left side) to during period of the COVID-19 pandemic (right side) among those reporting change in health or social services and using the respective health services before the pandemic. Weekly follow-up during the pandemic is labelled with deep blue, monthly follow-up during the pandemic is labelled with light blue, and less frequent follow-up during the pandemic is labelled with orange.


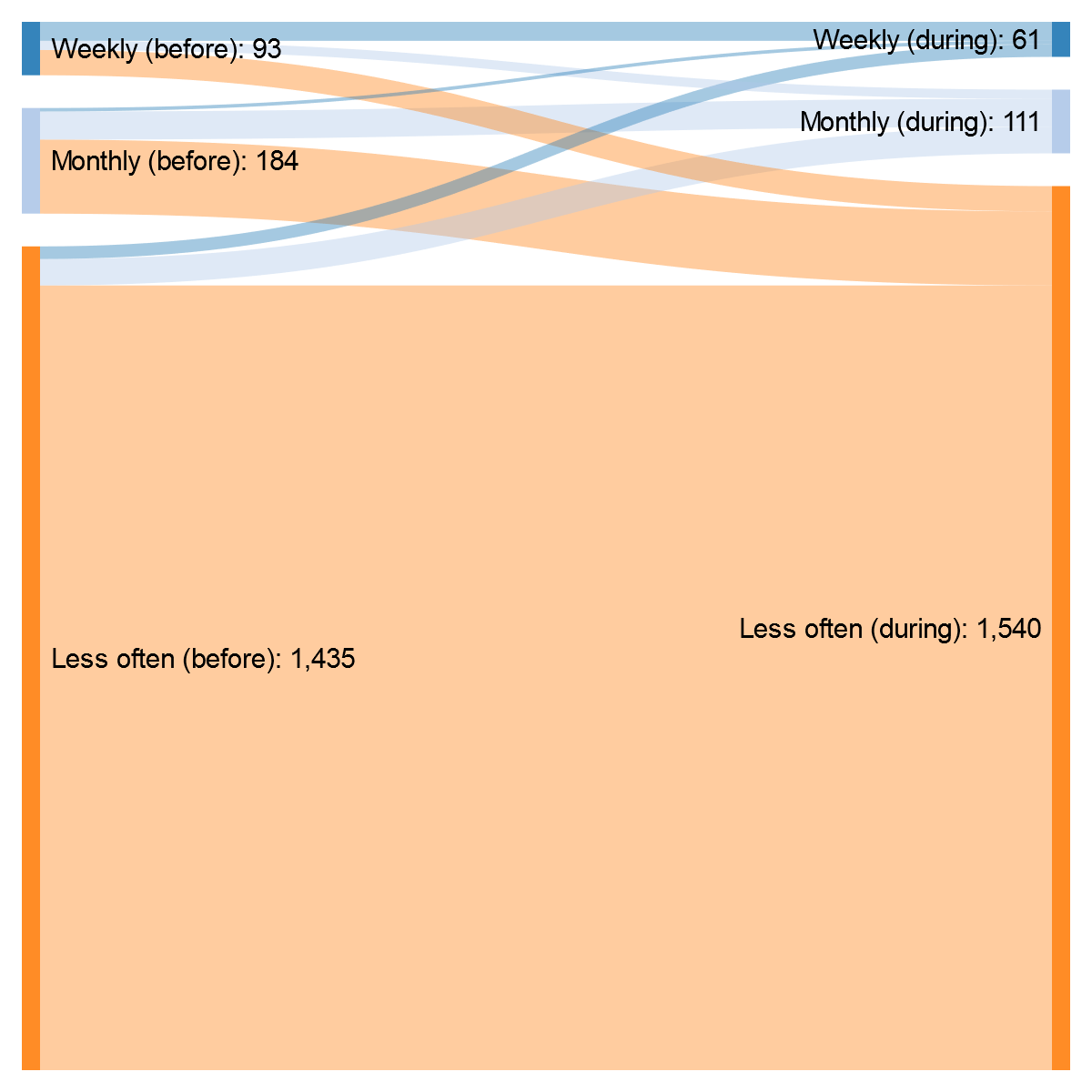


* p < 0.001 for all changes

**Figure S4:** Change in use of health services from home psychologists or physiotherapy (left side) to during period of the COVID-19 pandemic (right side) among those reporting change in health or social services and using the respective health services before the pandemic. Weekly follow-up during the pandemic is labelled with deep blue, monthly follow-up during the pandemic is labelled with light blue, and less frequent follow-up during the pandemic is labelled with orange.


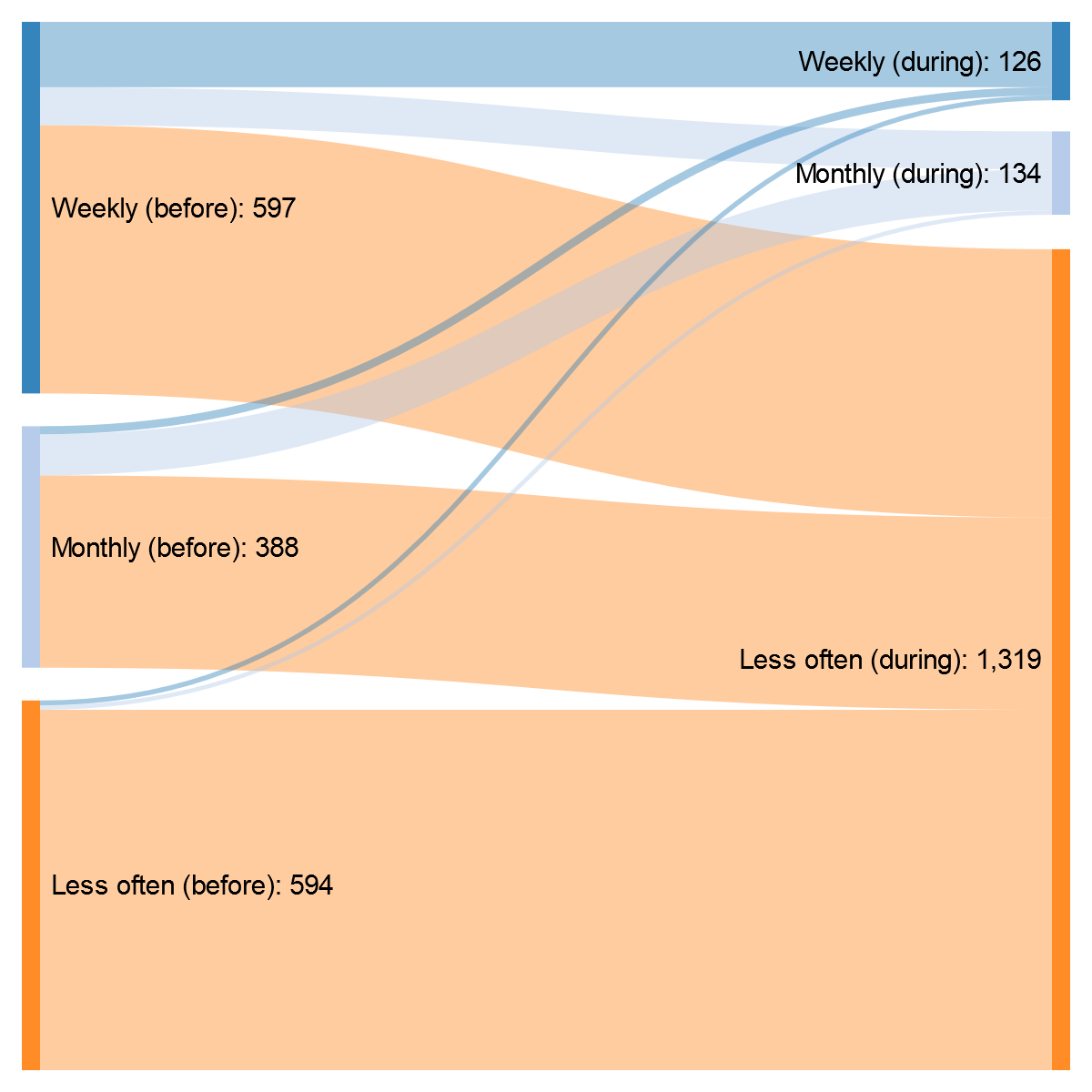


* p < 0.001 for all changes

**Figure S5:** Change in use of health services from home mental health services for children and adolescents (left side) to during period of the COVID-19 pandemic (right side) among those reporting change in health or social services and using the respective health services before the pandemic. Weekly follow-up during the pandemic is labelled with deep blue, monthly follow-up during the pandemic is labelled with light blue, and less frequent follow-up during the pandemic is labelled with orange.


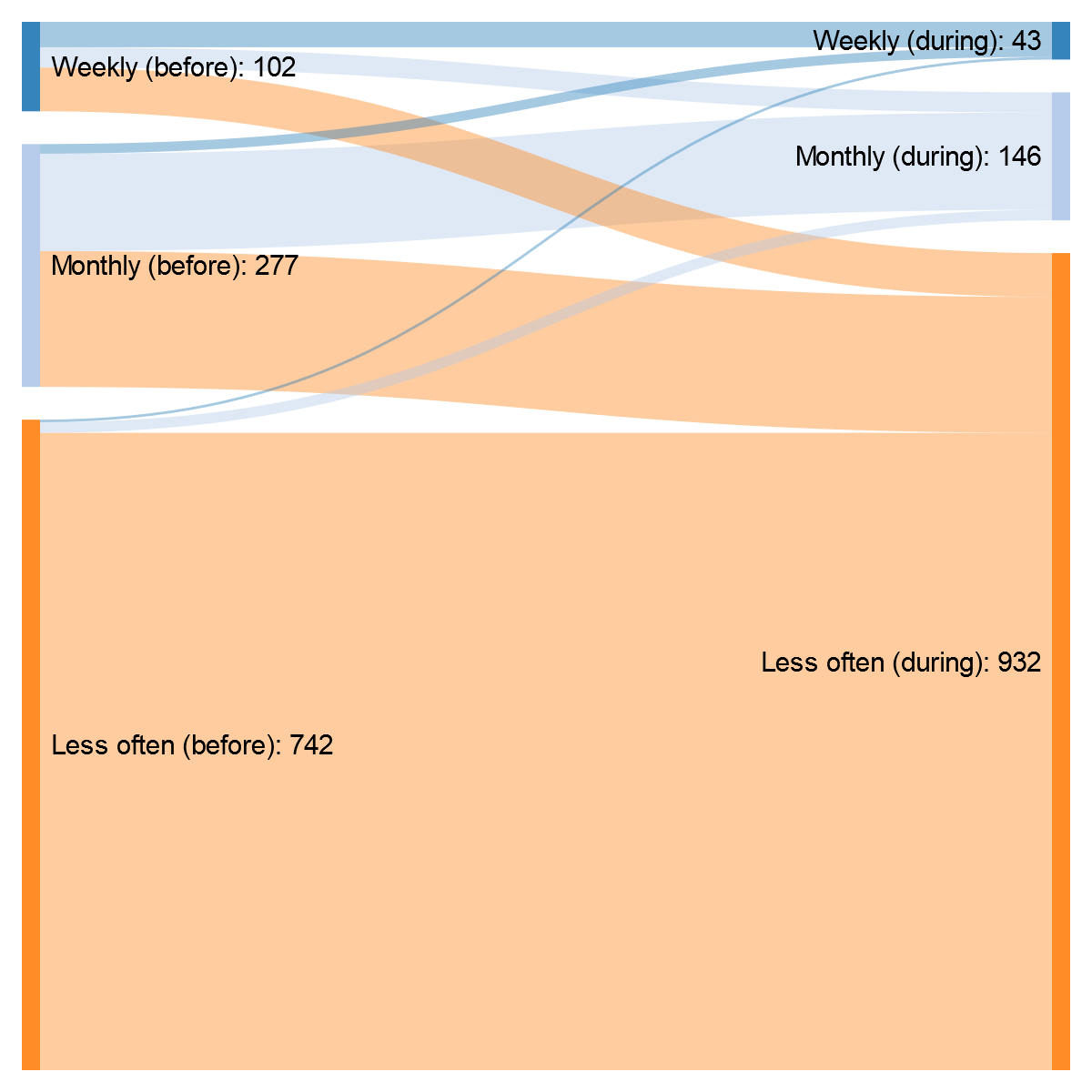


* p < 0.001 for all changes

**Figure S6:** Change in use of other health services (left side) to during period of the COVID-19 pandemic (right side) among those reporting change in health or social services and using the respective health services before the pandemic. Weekly follow-up during the pandemic is labelled with deep blue, monthly follow-up during the pandemic is labelled with light blue, and less frequent follow-up during the pandemic is labelled with orange.


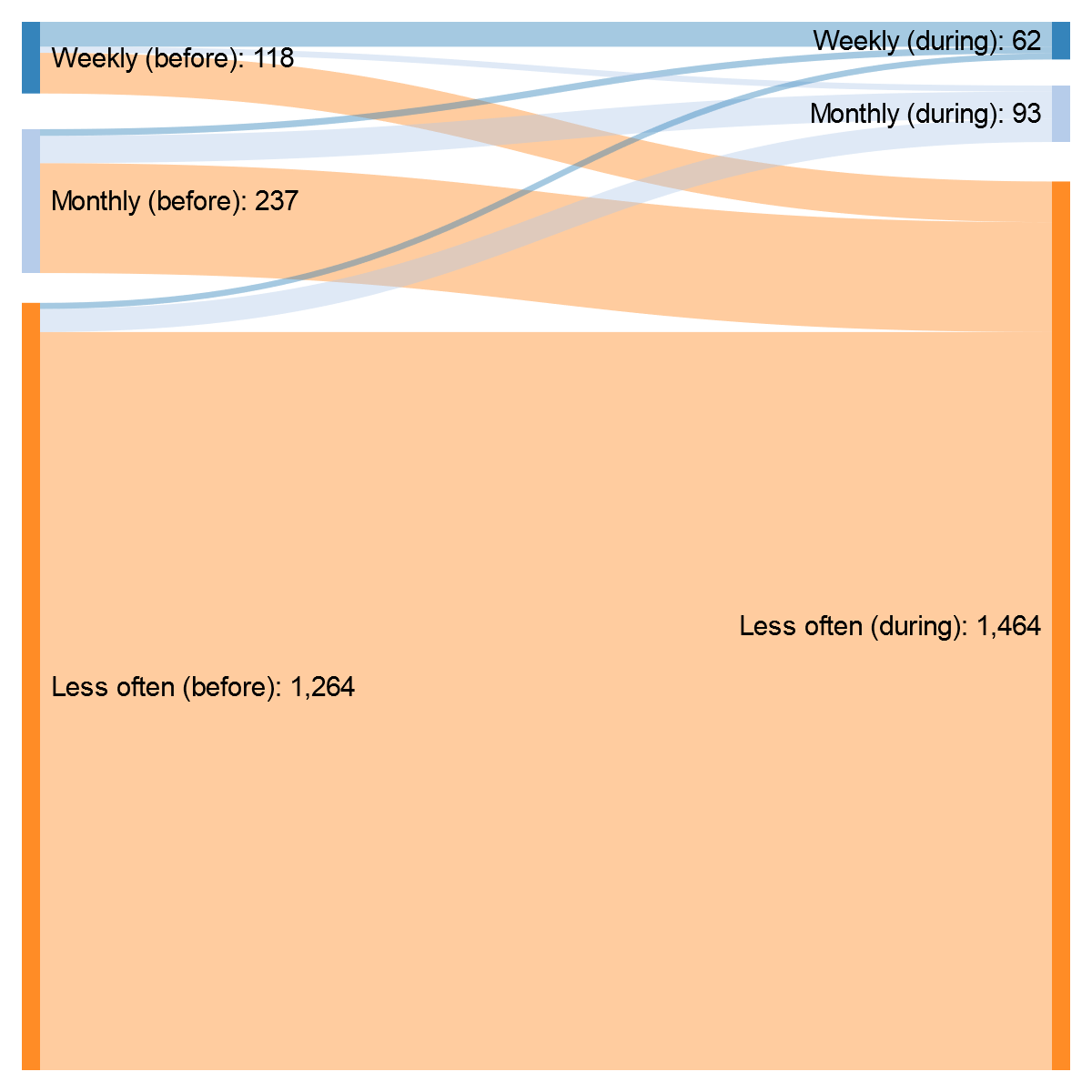


* p < 0.001 for all changes

**Figure S7:** Combined figure for change in use of various health services from before (left side) to during period of the COVID-19 pandemic among those reporting change in health or social services and using the respective health services before the pandemic. Weekly follow-up during the pandemic is labelled with deep blue, monthly follow-up during the pandemic is labelled with light blue, and less frequent follow-up during the pandemic is labelled with orange. Following services are presented: 1. General practitioner services (top left), 2. Home nursing (top middle), 3. Health services from hospital (top right), 4. Psychologists or physiotherapy (bottom left), 5. Other health services (bottom middle), 6. Mental health services for children and adolescents (bottom right).


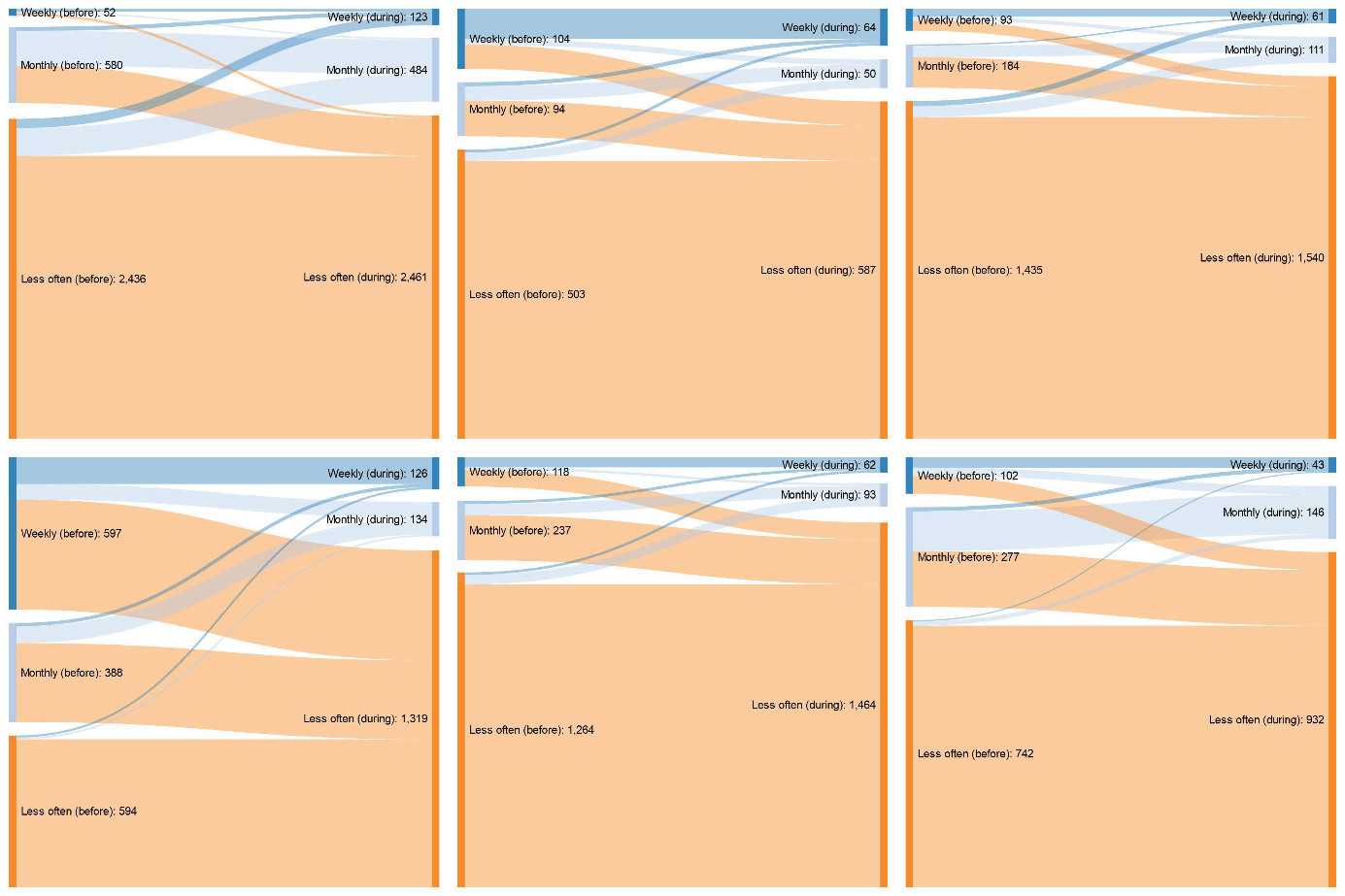


* p < 0.001 for all changes
